# The ever wider clinical spectrum of *RMND1*-related disorders and limitedness of phenotype-based classifications

**DOI:** 10.1007/s00109-023-02356-x

**Published:** 2023-08-16

**Authors:** Alexis V. Rioux, Nicolas AD. Bergeron, Julie Riopel, Nicolas Marcoux, Catherine Thériault, Peter V. Gould, Alexandre P. Garneau, Paul Isenring

**Affiliations:** 1 CHU de Québec, Service of Nephrology, Faculty of Medicine, Université Laval, QC G1R 2J6 Québec, Canada; 2 CHU de Québec, Service of Pathology, Faculty of Medicine, Université Laval, Québec, QC G1R 2J6 Canada; 3 CHU de Québec, Service of Hematology, Faculty of Medicine, Université Laval, Québec, QC G1R 2J6 Canada; 4 Service de Néphrologie–Transplantation Rénale Adultes, Hôpital Necker‑Enfants Malades, AP‑HP, Inserm U1151, Université Paris Cité, rue de Sèvres, Paris, France

**Keywords:** RMND1, Perrault syndrome, Mitochondriopathy, Myelodysplasia, Myopathy

## Abstract

**Abstract:**

*RMND1* has been identified as a mitochondriopathy-associated gene less than 12 years ago. The most common phenotype related to this gene is an early onset, severe form of encephalomyopathy that leads to death in a medium time of three years after birth. However, milder and later onset presentations have been reported in some individuals, including two in whom the mitochondriopathy was identified at ~ 40 years of age, and the early onset presentations have been the object of no reports in those who survived beyond age 10. It is thus unclear how lethal *RMND1*-related conditions really are. We herein describe the oldest case to have been identified hitherto with this condition, i.e., that of a white female who was 61 at the time of diagnosis but was still active in her everyday life. The gene defect identified was nonetheless associated with many manifestations including ovarian insufficiency and sensorineural hearing loss (two features of what is currently designated as Perrault syndrome) as well as chronic renal failure, asymptomatic myopathy, leukopenia, and a few others. In our opinion, this case is of great translational interest for at least three reasons. First, it hints towards the possibility of near-normal life expectancies in some if not many individuals with RMND1 insufficiency. Second, it underlines the wide clinical spectrum associated with this gene. Third, it brings us to question the use of eponyms and syndromic features to identify the true etiology of multisystemic phenotypes.

**Key messages:**

- *RMND1*-related conditions typically manifest at an early age with a progressive and lethal form of encephalomyopathy.
- More benign presentations have been described with some being categorized as Perrault syndrome but none have been diagnosed after the age of 45.
- The clinical spectrum and presenting age of *RMND1*-related mitochondriopathies are probably much more varied than implied in the current literature.
- The case reported in this manuscript illustrates the limitedness of phenotype-based classifications of genetic disorders to identify the defect at cause.

## Introduction

Biallelic mutations in a protein called Required for Meiotic Nuclear Division Protein 1 (RMND1) have been identified as a cause of infantile encephalomyopathy in 2012 and additional clinical phenotypes afterwards [1, 2]. This protein is known to be expressed in the mitochondria where it acts as a stabilizer of ribosomes near mRNA maturation sites [1, 2] and is aptly recognized as an active player in the process of oxidative phosphorylation [3].

**Fig. 1:**
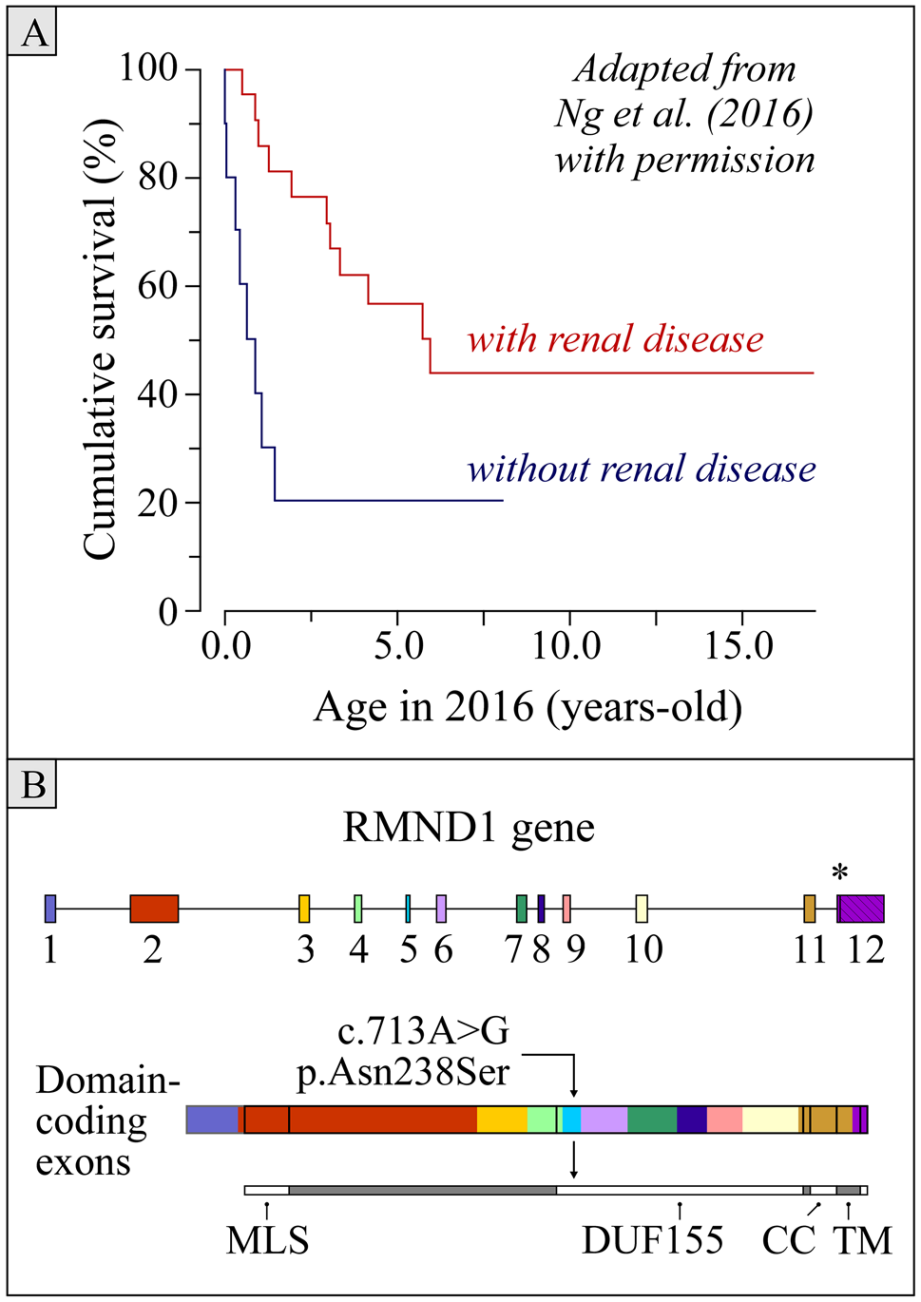
Cumulative survival of individuals with mutations in *RMND1*. **A** Kaplan–Meier survival curves with and without renal failure. Reproduced from Ng et al*.* [4]. **B** RMND1. Top row, *RMND1* gene with exon–intron organization. Middle and bottom rows, encoded protein showing domain–exon relationships. Abbreviations and signs: CC, coiled coil domain; DUF, domain of unknown function; MLS, mitochondrial localization signal; TM, transmembrane domain; *, stop codon. Variant identified in the current case is just past the middle of exon 5 as pointed by the arrow. Length of exons, introns, and domains are drawn to scale. Genes tested in the *Renome* are listed in Footnote

In a series of 32 cases from Ng et al. in 2016 [4], *RMND1*-related phenotypes manifested before age 2 in all children and were associated with very low survival rates (see Fig. 1A). Clinical findings varied in severity, but typically ranged from pronounced encephalomyopathy to slowly progressive and multisystemic abnormalities including sensorineural deafness and developmental delay. Interestingly, chronic kidney disease (CKD) was also found to be a related manifestation but associated with better survival rates (Fig. 1A).

Certain *RMND1* mutations have been recently linked to milder phenotypes. In this regard, Ozieblo et al. [5] uncovered new pathogenic variants in two sisters who were around 40 at the time of evaluation and known to be affected by sensorineural hearing loss and ovarian failure (Perrault syndrome) with CKD. One of these variants (p.Gly195Arg) was from a gene defect in exon 3 and the other (p.Tyr273Ser) from a gene defect in exon 6 (see Fig. 1B). Of note, both sisters exhibited no neurological deficits and had completed high-degree educations.

In general terms, the classification of genetic diseases based on phenotypes [6] may represent a source of bias as it is not etiology oriented. For instance, Perrault syndrome has been associated with 8 different loci and can occur as an isolated condition or in the setting of multisystemic dysfunctions [7]. The same applies to mitochondrial disorders and perhaps even more so given that the same phenotype can be associated with a variety of genetic defects and the same genetic defect with a variety of phenotypes.

The following report illustrates this potential caveat eloquently. Additionally, we have identified a multitude of clinical abnormalities that transgress the established phenotypic boundaries of Perrault syndrome and that are more in line with a multisystemic form of mitochondriopathy. The following report is also the first one to describe a case *RMND1*-related disorder that came to be diagnosed in a sexagenarian.

## Reason of consultation

The patient was referred to our nephrogenetic clinic for pre-renal transplantation evaluation in the setting of several medical conditions of unknown causes. She was a white female aged 61 at the time of evaluation and followed by a predialysis care clinic. The other conditions were sensorineural hearing loss, ovarian failure, and elevated serum creatine kinase (CK) levels of long-standing duration as well as mild leukopenia, osteoporosis, and skin cancers of more recent onset.

## Materials and methods

### Chart reviews and clinical assessment

The patient was evaluated by reviewing her medical history based on the numerical archives of five different hospitals and was subjected to a routine medical inquiry as well as physical examination. At the time of consultation, she had two brothers who were also evaluated through a questionnaire.

### Paraclinical assessment

#### Germinal DNA testing

Genetic testing was carried out through next-generation targeted exome sequencing with a homemade DNA panel termed *Renome* (see the genes included in Footnote). At the time of analysis, this panel was comprised of 225 genes that had been (tentatively or definitely) linked to chronic renal failure. It included several nuclear-encoded mitochondrial genes.

Almost all nucleotides (99.22%) within the genes assayed were sequenced at a coverage of more than 20x. Copy number variations were also looked for through next-generation exome sequencing at the same standard coverage and at a resolution of ~ 2 exons [8, 9]. The variants identified were classified as pathogenic, likely pathogenic, risk allele, of unknown significance, likely benign or benign as per American Genetic Association standards [10].

#### Muscle biopsy

The patient underwent a quadricipital muscle biopsy to investigate the increase in CK levels. Sections of the specimen collected were subjected to myosin heavy chain fast (MHCF), succinate dehydrogenase (SDH), and cytochrome C oxidase (COX) stains after which they were analyzed under light microscopy. An ultrastructural study could not be obtained but was carried out in the bone. Note that the MHCF stain detects type II muscular fibers specifically.

#### Medullar aspiration and biopsy

An osteomedullary biopsy with bone marrow aspiration was also ordered given the mild leukopenia of recent onset. The specimens were subjected to hematoxylin and eosin (H&E), Giemsa, and Prussian blue stains after which they were analyzed by light microscopy. It is on the osteomedullary biopsy that the ultrastructural evaluation was conducted to characterize the morphology of the mitochondria.

## Results

### Chart review and physical assessment

#### Chart review

**Fig. 2:**
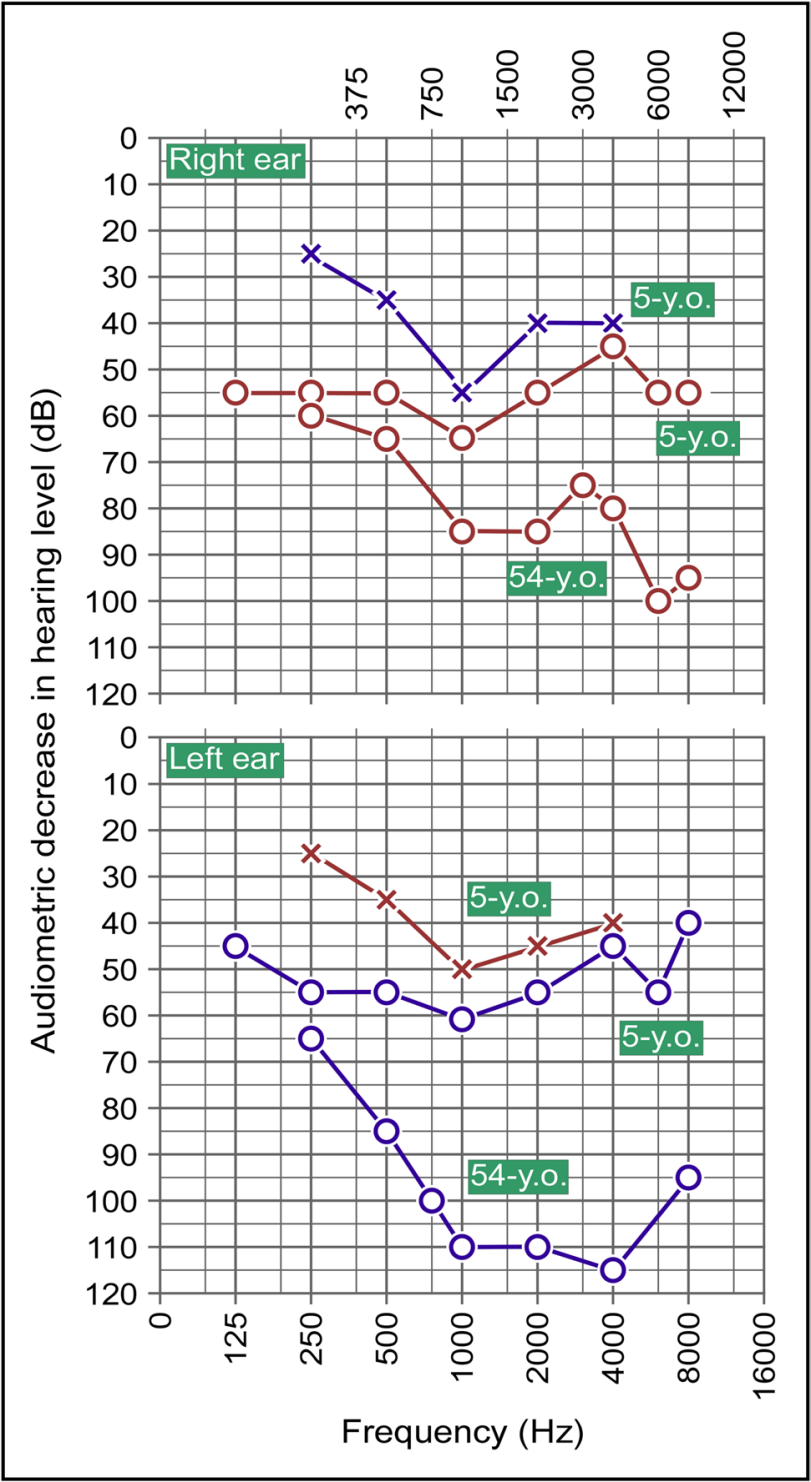
Audiograms taken at age of 5 and 54. Top panel, right ear. Bottom panel, left ear. Signs: cross, bone conduction; circle, air conduction

The hearing loss had been first noticed at age 3 and was slightly asymmetrical initially. Based on Fig. 2, bone conduction (× signs) at that time was found to be decreased by 20 to 55 dB among all frequencies. Air conduction (Ο signs) was also abnormal but attributed to repetitive otitis media. Hearing loss worsened steadily and eventually became symmetrical (Fig. 2) and the difference in air and bone conduction disappeared (not shown).

As for the ovarian insufficiency, it had brought the patient to consult at age 16 for failure to menstruate even once. Physical development was then Tanner stage III, indicating a slight delay in sexual maturation. Investigations pointed towards primary XX amenorrhea by showing increased serum levels of FSH and LH, an ovarian strip upon laparoscopic evaluation, and a normal karyotype (not shown).

**Fig. 3:**
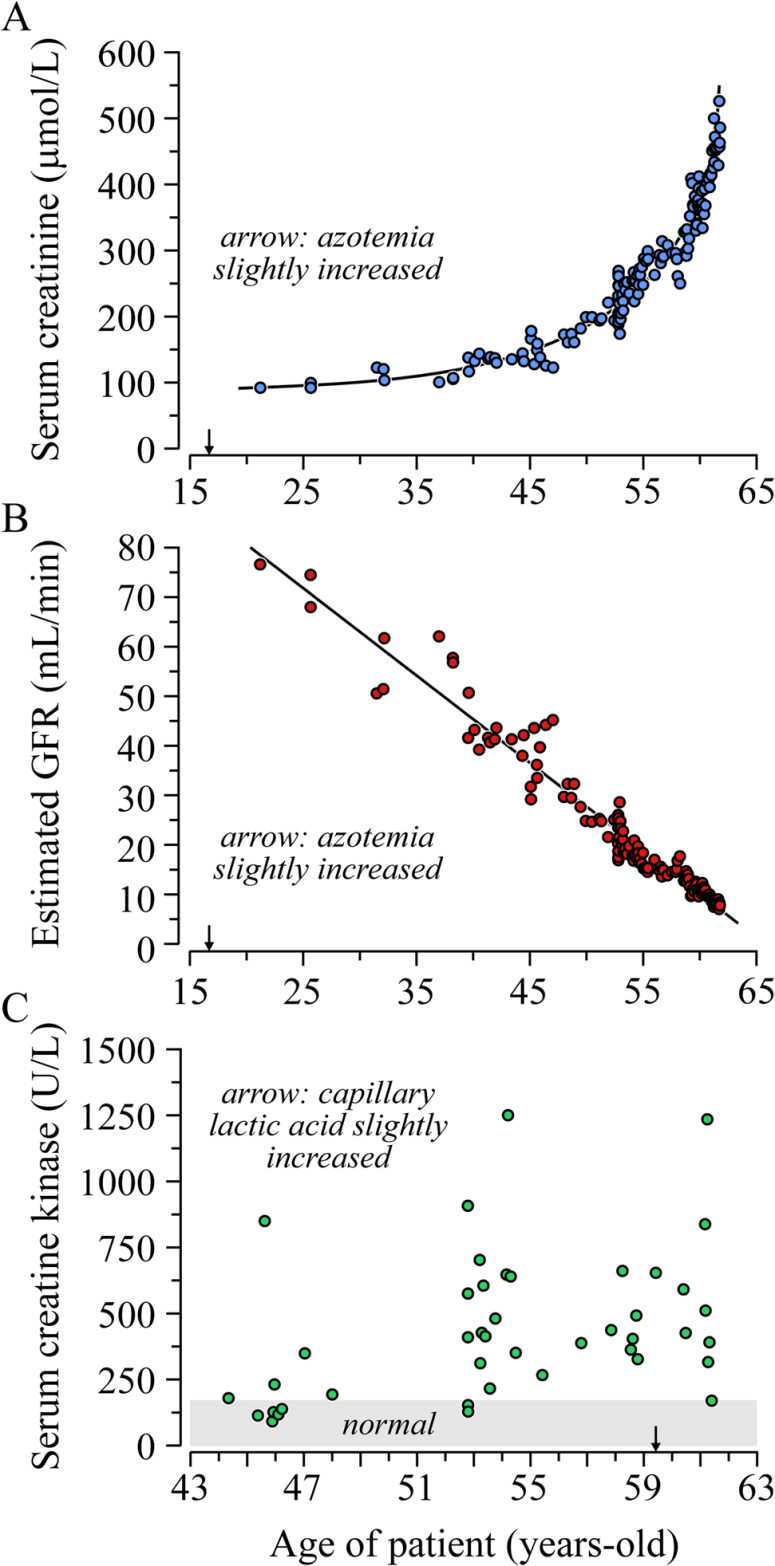
Laboratory results prior to consultation. As a function of age are shown: **A** serum creatinine, **B** estimated GFR, and **C** serum CK levels. Arrows point to slightly increased azotemia (33 mg/dL) or capillary lactate levels (2.6 mmol/L) at the ages shown

Renal failure on its part had progressed linearly for over 40 years (Fig. 3A, B). At age 21, estimated glomerular filtration rate (the first available) was 76 mL/min (based on a creatinine of 92 µmol/L) and declined steadily afterwards in the absence of hematuria or proteinuria. At age 24, an ultrasound revealed mild kidney atrophy and a renal biopsy no abnormalities under light microscopy by routine staining and immunofluorescence.

As for the serum CK levels, they had been found to be increased for the first time at age 44. As shown in Fig. 3C, they were subsequently measured on multiple occasions and seen to be elevated most of the time. An autoimmune profile (including anti-Jo1 antibodies) was found to be normal at age 54. The possibility of Perrault syndrome was evoked then by a consultant but considered unlikely because of the muscle disorder and the other abnormalities.

The other problem of more recent onset was the leukopenia as it had been first noted when the patient reached her mid-fifties. White blood cells were then in the lower limit of normal (at ~ 5.0 × 10^9^/L) in the absence of morphological abnormalities. During the ten months that preceded the consultation in nephrogenetics, they had decreased to a mean of 3.5 × 10^9^ /L. No abnormalities were observed in the peripheral red blood and platelet lineages.

Besides the leukopenia, additional conditions of relatively recent onset had also been identified. They were as follows: ectasia of the ascending aorta that was still mild not long before the genetic evaluation, osteoporosis that was already moderate when first assessed, and a total of 10 squamous or basal cell carcinomas of the skin that occurred at different locations between age 55 and 61.

#### Clinical assessment

When the patient was evaluated, she was on a normal CKD pharmacopeia including Darbepoetin. She had described herself as being active, having good tolerance to efforts, and indulging in healthy habits. She also had no history of excessive sun exposure. Physical examination was otherwise unremarkable with no overt stigmata of a syndromic disorder and with a type 3 skin complexion.

**Fig. 4:**
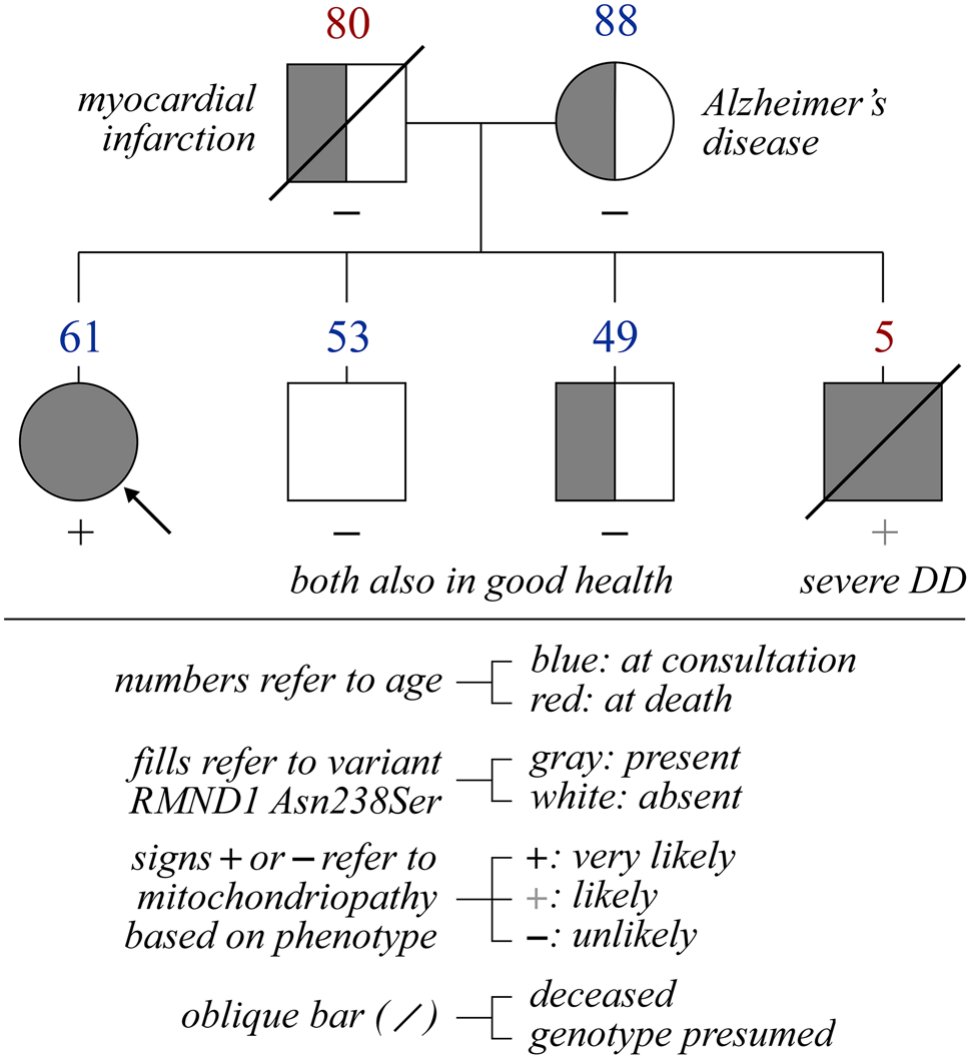
Pedigree of the proband’s first-degree relatives. Refer to bottom note in the figure for signification of colors and signs. Abbreviations: DD, developmental delay

A pedigree of the patient’s family obtained at the time of consultation is shown in Fig. 4. As seen, the mother was then 88 and affected by Alzheimer’s disease, the father died at age 80 of myocardial infarction (but had no other conditions), one brother died at age 5 while suffering from advanced developmental disability, and two middle-aged brothers were in good health.

### Paraclinical assessment

#### Germinal genetic testing

The analysis carried out showed that the patient was homozygous for the pathogenic *RMND1* (NM_017909.3) c.713A > G (p.Asn238Ser) variant. As shown in Fig. 1B, the defect identified is located in one of the DUF155-encoding exons, i.e., near the middle of exon 5 [2]. It is present in the general population at a maximal allele frequency of 0.04% and its physicochemical distance relative to the native residue translates into a Grantham score of 46.

This variant was looked for in the brothers and mother of the proband through site-specific testing. As shown in Fig. 4, it is seen to affect one allele in both the mother and younger brother but neither of the alleles in the older brother. Based on these findings and the phenotype exhibited by each family member, we concluded that the father was a carrier of the gene defect and that the deceased brother was affected by a *RMND1*-related disorder.

#### Muscle biopsy

**Fig. 5:**
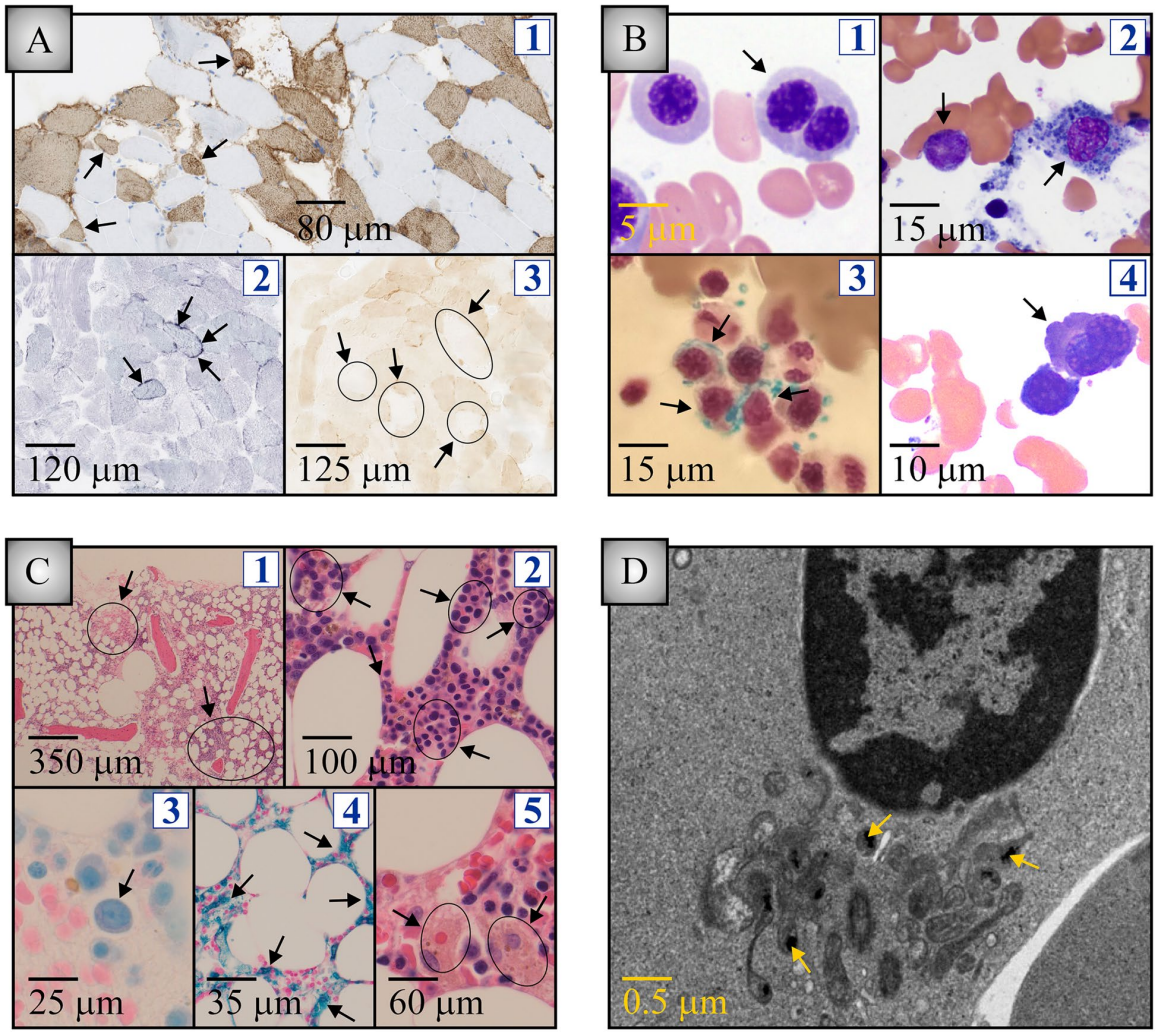
Histological analysis of muscle and bone marrow sections. Abnormalities are shown through arrows. Stain used in this legend are between parentheses. **A** Muscle biopsy. Subpanel 1 (MHCF): atrophied muscle fibers. Subpanel 2 (COX): subsarcolemmal aggregates representing apoptotic mitochondria. Subpanel 3 (SDH): fibers devoid of enzyme activity. **B** Bone marrow aspirate. Subpanel 1 (Giemsa): binucleated erythroblast. Subpanel 2 (Giemsa): erythroblast with scant cytoplasm (left) and hemosiderophage (right). Subpanel 3 (Prussian blue): islet of ring sideroblasts. Subpanel 4 (Giemsa): abnormally small megakaryocyte. **C** Bone marrow biopsy. Subpanel 1 (H&E): area of hypocellularity (left) and hypercellularity (right). Subpanel 2 (H&E): areas densely populated with erythroblasts. Subpanel 3 (Giemsa): megaloblastoid cell. Subpanel 4 (Prussian blue): diffuse iron deposition. Subpanel 5 (H&E): erythrophagocyte (left) and hemosiderophage (right). Note that a cytogenetic analysis carried out on 26 mitoses revealed normal. **D** Electron microscopy of a sideroblast. Iron deposition is seen in several of the mitochondria captured

The abnormalities seen were indeed consistent with a mitochondrial disorder. For instance, and as pointed by arrows in the subpanels (S1 to S3) of Fig. 5A, a certain number of fibers were found to be atrophied (S1), to contain subsarcolemmal aggregates likely representing apoptotic mitochondria (S2) and to be completely devoid of SDH activity (S3).

#### Medullar aspiration and biopsy

On the aspirate, several morphological abnormalities were also noted. Examples of the observations made are shown through arrows in the subpanels (S1 to S4) of Fig. 5B. They are as follows: a binucleated erythroblast (S1), a cytoplasm-free erythroblast on the left and a hemosiderophage on the right (S2), ring sideroblasts (S3), and a micromegakaryocyte (S4). Note that 7% of erythroblasts contained iron deposits.

As for the biopsy, it also revealed myelodysplastic changes. Illustrations of the abnormalities seen are shown through arrows in the subpanels (S1 to S4) of Fig. 5B. They are as follows: area of low cellularity on the left and high cellularity on the right (S1), erythroblast-rich islets (S2), a megaloblastoid cell (S3), global iron overload (S4), and an erythrophagocyte on the left with hemosiderophage on the right (S5). Note that the number of hemosiderophages was high.

Lastly, the ultrastructural evaluation of the bone marrow confirmed the presence of iron deposits in the mitochondria of sideroblasts (see examples pointed by arrows in Fig. 5D). On the image obtained, additionally, these organelles displayed a frankly abnormal appearance in the form of nanotunnels but they were not seen to be oddly distributed within the cytoplasm.

## Discussion

In the current report, we described the case of a 61-year-old female who was affected by a progressing multisystemic condition and subjected to an in-depth investigation. This condition began to manifest with hearing loss early in life, ovarian and renal failure during teenagerhood, and increased serum CK levels during middle age. The data obtained led to the conclusion that such conditions could be attributed to a *RMND1*-associated mitochondriopathy.

A remarkable aspect of our case is that the patient was 61 when diagnosed but still active in her everyday activities. She would thus be the oldest individual and among the healthiest ones to be affected by an *RMND1*-related condition. As mentioned, we have come across only two case reports of analogous presentations but in patients who were much younger than ours when they were investigated [5]. Otherwise, individuals with this condition rarely live beyond age 10 [4].

Another interesting aspect of this case is that a diagnosis of Perrault syndrome had been evoked at one point but considered unlikely in the end as it would have failed to account for several of the manifestations [11]. Yet, a few cases of Perrault syndromes have now been reported to have developed chronic renal disease after the hearing loss or ovarian failure were first noticed [12].

This case illustrates the limitations of classifying genetic disorders on the basis of nosological considerations. In particular, it is well known that the same mutation in a given gene can translate into a wide constellation of clinical presentations [13]. Evaluating patients according solely to phenotype can lead one to exclude the culprit as certain clinical features are lacking or are not supposed to be part of the classical presentation.

This caveat is of particular importance in mitochondriopathies for both the nuclear and mitochondrial forms given that the genetic background of mitochondria is normally heteroplasmic among most cell types to start with [14]. It is perhaps one of the reasons why the same RMND1 variant (e.g., p.Asn238Ser) has been associated with such diverse presentations [4] and why the more recently identified *RMND1* variants (e.g., p.Gly195Arg and p.Tyr273Ser) should not be given a prognostic meaning too early on [5].

The less severe forms of *RMND1*-related conditions often manifest as slowly evolving oligo or multisystemic disorders [5, 15]. In four individuals, the presentations seen were those of Perrault syndrome associated with renal failure (as mentioned) or white matter encephalopathy [15]. Interestingly, none of these individuals were reported to be affected by the other conditions that were seen in our patient but they were again much younger.

Among these other conditions were the myelodysplastic syndrome and skin cancers that we considered unusual in presentations and unexpected for an individual in her mid-fifties. In addition, one must remember that mitochondrial disorders have been associated with sideroblastic anemia [16] and that healthy mitochondria play a key protective role against skin cancers and photo aging [17]. It is thus tempting to postulate that the defect in *RMND1* accounted for both these conditions.

Two other conditions observed were the moderate form of osteoporosis and mildly ectatic ascending aorta. Whether they could also be manifestations of the gene defect in *RMND1* is obviously less certain. However, mitochondrial disorders can cause any tissues to become dysfunctional and have even been associated with premature bone loss [18, 19] and aortopathies [20, 21] in a number of individuals.

In conclusion, this case should convey the idea that it is perhaps time to abandon the eponym- or acronym-based classification of mitochondriopathies as this categorization scheme comes with too much overlap or variability among the clinical entities that it calls for. Why not resort to a simple nomenclature that points to the culprit as a modern or precise approach of terming genetic disorders? In our case, could the culprit identified explain it all?

## Footnote

*Renome*: *ABCC6, ACE, ACTN4, ADAMTS13, AGT, AGTR1, AGXT, AHI1, ALG1, ALMS1, AMN, ANLN, ANOS1, APOE, APOL1, APRT, ARHGAP24, ARHGDIA, ARL6, ATXN10, BBS1, BBS12, BBS2, BMP4, BMP7, C3, C3AR1, C4BPA, C4BPB, C5, C5AR1, C6, C7, C8A, C8B, C8G, C9, CC2D2A, CD151, CD2AP, CD46, CD55, CD59, CDC5L, CEP164, CEP290, CEP41, CEP83, CFB, CFD, CFH, CFHR1, CFHR2, CFHR3, CFHR4, CFHR5, CFI, CFP, CHD1L, CLCN5, CLDN16, CLDN19, COL4A3, COL4A4, COL4A5, COQ2, COQ6, COQ8B, CPT2, CR1, CR2, CRB2, CTNS, CUBN, DACT1, DCDC2, DGKE, DSTYK, EHHADH, ELMO1, ELN, EMP2, ENPP1, ERCC4, EXT1, EYA1, F12, FAN1, FANCB, FAT1, FGA, FGF20, FGFR2, FKRP, FN1, FOXC2, FOXP3, FRAS1, FREM1, FREM2, GAA, GATA3, GLA, GLI3, GLIS2, GRHPR, GRIP1, HNF1B, HOGA1, HOXA13, HOXA4, HOXB6, HPSE2, IFT122, IFT140, IFT43, INF2, INVS, IQCB1, ITGA3, ITGA8, ITGB4, KANK1, KANK2, KANK4, KYNU, LAGE3, LAMB2, LMX1B, LPL, LRIG2, LRP2, LRP4, LZTFL1, MAFB, MAGI2, MAPKBP1, MEFV, MGME1, MMACHC, MOCOS, MRPS7, MTOR, MUC1, MUT, MYH9, MYO1E, NEIL1, NEK8, NIPBL, NPHP1, NPHP3, NPHP4, NPHS1, NPHS2, NRIP1, NUP107, NUP160, NUP205, NUP93, NXF5, OCRL, OSGEP, PAX2, PBX1, PDSS2, PLCE1, PLG, PMM2, POLG, PTPRO, PYGM, REN, RET, RMND1, ROBO2, RPGRIP1L, RRM2B, SALL1, SARS, SARS2, SCARB2, SDCCAG8, SEC61A1, SGPL1, SIX1, SIX2, SIX5, SLC22A12, SLC22A5, SLC2A9, SLIT2, SMARCAL1, SOX17, SRGAP1, ST3GAL1, TBX18, TGFB1, THBD, TMEM126B, TMEM138, TMEM216, TMEM67, TP53RK, TPRKB, TRAP1, TRPC6, TSC1, TTC21B, TTC8, UMOD, UPK3A, VTN, VWF, WDR19, WDR34, WDR35, WDR73, WIF1, WNT4, WT1, XPNPEP3, XPO5, ZAP70, ZMPSTE24.*

## Data Availability

Data will be made fully available upon request.
